# COMT Inhibition Alters Cue-Evoked Oscillatory Dynamics during Alcohol Drinking in the Rat

**DOI:** 10.1523/ENEURO.0326-18.2018

**Published:** 2018-10-31

**Authors:** A. M. McCane, S. Ahn, L. L. Rubchinsky, S. S. Janetsian-Fritz, D. N Linsenbardt, C. L. Czachowski, C. C. Lapish

**Affiliations:** 1 Department of Psychology, Indiana University Purdue University Indianapolis, IN 46202; 2 Department of Mathematics, East Carolina University, NC 27858; 3 Department of Mathematical Sciences, Indiana University Purdue University Indianapolis, IN 46202; 4 Stark Neuroscience Research Institute, Indiana University School of Medicine, Indianapolis, IN 46202

**Keywords:** alcohol preferring rat, tolcapone, θ oscillation, prefrontal cortex, nucleus accumbens, alcoholism, alcohol use disorder

## Abstract

Alterations in the corticostriatal system have been implicated in numerous substance use disorders, including alcohol use disorder (AUD). Adaptations in this neural system are associated with enhanced drug-seeking behaviors following exposure to cues predicting drug availability. Therefore, understanding how potential treatments alter neural activity in this system could lead to more refined and effective approaches for AUD. Local field potentials (LFPs) were acquired simultaneously in the prefrontal cortex (PFC) and nucleus accumbens (NA) of both alcohol preferring (P) and Wistar rats engaged in a Pavlovian conditioning paradigm wherein a light cue signaled the availability of ethanol (EtOH). On test days, the catechol-o-methyl-transferase (COMT) inhibitor tolcapone was administered prior to conditioning. Stimulus-evoked voltage changes were observed following the presentation of the EtOH cue in both strains and were most pronounced in the PFC of P rats. Phase analyses of LFPs in the θ band (5–11 Hz) revealed that PFC-NA synchrony was reduced in P rats relative to Wistars but was robustly increased during drinking. Presentation of the cue resulted in a larger phase reset in the PFC of P rats but not Wistars, an effect that was attenuated by tolcapone. Additionally, tolcapone reduced cued EtOH intake in P rat but not Wistars. These results suggest a link between corticostriatal synchrony and genetic risk for excessive drinking. Moreover, inhibition of COMT within these systems may result in reduced attribution of salience to reward paired stimuli via modulation of stimulus-evoked changes to cortical oscillations in genetically susceptible populations.

## Significance Statement

Alcoholism is highly heritable and genetic vulnerability is associated with increased likelihood of alcohol use-related problems. Presentation of environmental stimuli paired with alcohol are capable of inducing craving and relapse in alcohol-dependent individuals. The work described here using a rodent model of cued ethanol (EtOH) availability suggests that altered corticostriatal activity may be associated with genetic vulnerability to abuse alcohol. Additionally, our data suggest that inhibition of the catechol-o-methyl-transferase (COMT) enzyme activity may reduce the influence that alcohol-conditioned stimuli (CSs) elicit on behavior, resulting in a reduction of cued alcohol seeking in genetically susceptible individuals.

## Introduction

Environmental stimuli associated with drugs of abuse acquire motivational properties (Robinson and Berridge, 1993) capable of inducing drug craving (Myrick et al., 2004; Reid et al., 2006) and seeking (Katner et al., 1999; See, 2002). Exposure to these stimuli has been consistently shown to activate brain regions across the mesocorticolimbic (MCL) system, including the prefrontal cortex (PFC) and nucleus accumbens (NA; Grusser et al., 2004; Myrick et al., 2004; Oberlin et al., 2016). The activation of these structures by drug-paired cues (Schacht et al., 2013) is thought to reflect the neural processes required to assign salience to these cues (Berridge and Robinson, 1998; Di Chiara, 1999). Furthermore, altered PFC signaling to the NA is critical for the transition to compulsive drinking (Seif et al., 2013). The goal of the current study was to determine the influence of alcohol-associated cues on neural synchrony between the PFC and NA.

Theta oscillations (5–11 Hz, in the rat) have been shown to facilitate plasticity (Abbott, 1992; Huerta and Lisman, 1996; Chauvette et al., 2012), as well as cognitive functions such as, stimulus evaluation (Başar and Güntekin, 2008) and reward processing (Kamarajan et al., 2008; van Wingerden et al., 2010a). Perturbations in θ activity are also associated with alcohol use (De Bruin et al., 2004; Kamarajan et al., 2004, 2012; Jones et al., 2006b), exposure (Krause et al., 2002), and genetic vulnerability to alcohol dependence (Kamarajan et al., 2006; Rangaswamy et al., 2007; Andrew and Fein, 2010). Additionally, individuals with alcohol use disorder (AUD) show alterations in the P300 response (Cohen et al., 2002), which is evoked by exposure to salient environmental stimuli (Jones et al., 2006a) and is hypothesized to be driven by changes in delta and θ oscillations (Jones et al., 2006a). Changes in θ synchrony between MCL structures may, therefore, convey risk for excessive drinking and provide a target to develop novel therapies for AUDs.

Alcohol preferring (P) rats are a validated preclinical model of AUD (Murphy et al., 2002; Froehlich, 2010; Bell et al., 2017). P rats are selectively bred for alcohol preference and display a robust alcohol seeking and drinking phenotype compared with their progenitor strain, Wistar rats (Murphy et al., 2002). Similar to individuals with a positive family history (FH+) for AUD, P rats exhibit lower P300 amplitudes relative to non-P rats (Ehlers et al., 1999). P rats also exhibit reduced neural phase locking (Criado and Ehlers, 2010), which is similar to impairments in phase locking observed in long-term abstinent alcoholics (Andrew and Fein, 2010). Higher neural firing rates in the PFC of P rats versus control rats during a cued-alcohol task (Linsenbardt and Lapish, 2015) are also consistent with increases in alcohol-paired stimuli-evoked activity in the PFC of FH+ individuals and individuals diagnosed with an AUD (George et al., 2001; Myrick et al., 2004; Kareken et al., 2010; Cservenka and Nagel, 2012). These observed changes in neural activity of the P rat may be attributable to observed alterations in regulatory systems, such as reduced expression of the glutamate metabotropic receptor 2 (Zhou et al., 2013) and dopamine (DA) levels in PFC (Engleman et al., 2006). Collectively, these data support the use of the P rat as a translational tool to investigate the relationship between neurophysiological alterations and excessive drinking.

The catechol-o-methyl-transferase (COMT) enzyme metabolizes catecholamine’s including DA in cortical regions (Mazei et al., 2002; Morón et al., 2002). Using the COMT inhibitor tolcapone, we have observed reductions in cued ethanol (EtOH) consumption in P rats (McCane et al., 2014) and reinforcer seeking (McCane et al., 2018). Tolcapone enhances cued medial (m)PFC DA efflux (Lapish et al., 2009) and therefore may remediate mPFC DA deficits in P rats (Engleman et al., 2006). The current experiments measured changes in neural activity in the mPFC and NA during cued alcohol seeking. We hypothesized that differences in θ oscillations between P and Wistar rats would underlie differences in alcohol seeking behaviors and that tolcapone administration would normalize aberrant neural activity in the P rat.

## Materials and Methods

### Behavior

A total of 63 datasets were analyzed from 16 animals: P (*N* = 10, 37 datasets; Indiana University) and Wistar (*N* = 6, 26 datasets; Harlan) rats. All animals arrived from both respective outside vendors within the same week to ensure habituation and rearing conditions were identical for both strains. Animals were single housed, weighed ∼250–300 g at the start of experiment, maintained on a reverse light dark cycle and supplied with food and water *ad libitum*. All animals initially received four weeks of intermittent two bottle access for 20% EtOH to pre-expose animals to EtOH (McCane et al., 2014; Linsenbardt and Lapish, 2015). Immediately following this induction phase, animals began training in the two-way cued access protocol (2CAP) as described previously (McCane et al., 2014). Briefly, behavior was conducted in a two-compartment operant chamber equipped with two stimulus lights, and two retractable sippers, one on each side of the chamber. Illumination of a stimulus light (conditioned stimulus; CS) for 2 s was followed by a 1-s interstimulus interval and then 10-s access to 10% EtOH (unconditioned stimulus: US). Each session consisted of 40 CS/US pairings or trials, randomized between sides. All animals had a minimum of three weeks conditioning before electrophysiological recordings.

### Pharmacology

To assess the influence of COMT inhibition on corticostriatal network dynamics, animals were treated with tolcapone (30 mg/kg) or vehicle (counterbalanced) as described previously (McCane et al., 2014, 2018).

### Electrophysiology

**Figure 1.**
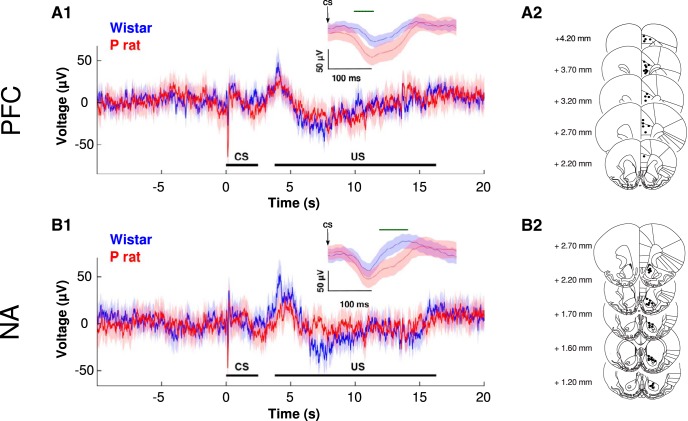
Voltage traces (1) and electrode placements (2) in the mPFC (***A***) and NA (*B*) during drinking trials. P rats (red) exhibit an augmented mPFC response to the CS (A1, inset) compared to Wistar rats (blue). All values are mean ± SEM. Bar depicts time when strains are different, *p* > 0.05.

**Figure 2.**
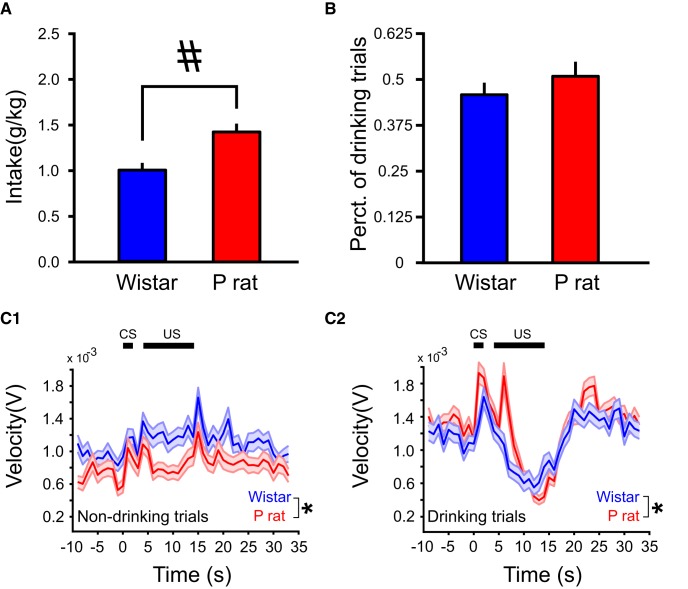
P rats consume more EtOH across sessions relative to Wistars (***A***) but do not differ in percentage of drinking trials (***B***). Overall movement velocities differ by strain for both drinking and non-drinking trials but both strains show a reduction in locomotor activity during drinking (***C2***) but not non-drinking trials (***C1***). All values are mean ± SEM; #*p* < 0.05, independent samples *t* test; *p < 0.05, main effect of strain.

Following conditioning, animals were anesthetized with isoflurane and implanted with custom-made electrophysiology probes. Probes were assembled using four 50-µm stainless-steel wires (California Fine Wires) fed through silica tubing and then secured to custom-made head caps via epoxy. Wires were externally referenced to a stainless-steel skull screw over the cerebellum. Probes were implanted over the mPFC (AP: 3.0, ML: 0.5, DV: -3.2) and NA (AP: 1.6, ML: 1.5, DV: -6.7; Fig. 1). Local field potentials (LFPs) were acquired with a 96 channel Neuralynx Cheetah recording system. LFPs were sampled at 32,556 Hz, amplified 2000 times, initially filtered between 0.01 and 1000 Hz, and then down sampled to 1017 Hz for analyses. Each session was recorded with video tracking software (Any-Maze) which was synchronized with the electrophysiological recoding system with submillisecond precision. This allowed the location of the animals to be recorded as *x*-, *y*-coordinates in separate voltage traces. These voltage traces were then used to compute the velocity of the animal’s movement (Fig. 2). Lastly, video recordings were manually scored to identify “drinking” versus “non-drinking” trials, that is, the animals were clearly vigilant and capable of observing the CS and then either consumed or did not consume the EtOH presented

### Power frequency analyses

**Figure 3.**
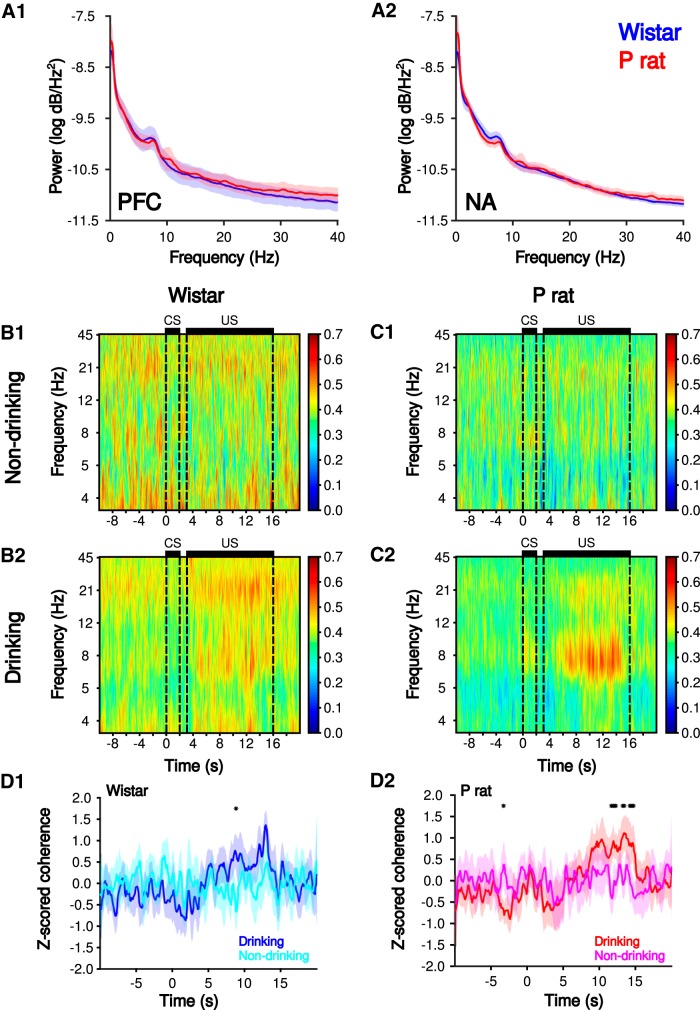
Theta oscillation are present in the 2CAP and are associated with drinking behaviors. Power spectral densities (***A***) in the mPFC (1) and NA (2) show a prominent peak in the θ frequency. Mean traces of mPFC-NA spectral coherence during non-drinking (1) and drinking (2) trials in Wistars (***B***) and P rats (***C***). Theta band wavelet coherence between the mPFC and NA for non-drinking (cyan, pink) and drinking (blue, red) trials in Wistars (***D1***) and P rats (***D2***); *p < 0.05 Bonferroni *post hoc*, drinking versus non-drinking.

Power spectral densities from the mPFC and NA were computed via multi-taper spectral decomposition from voltage traces extracted 10 s before, and 32 s after the CS. A 95% confidence interval was derived via *normfit.m* to test for significant differences between signals (Fig. 3*A*).

### Spectral coherence analyses

Wavelet coherence was computed using the *wcoher.m* function in MATLAB using Morelet wavelets smoothed over 20 ms. Wavelet coherence spectra were calculated on 30 s of the LFP signal extracted around the CS-fluid sequence (Fig. 3). For each animal, spectra were calculated for each trial, smoothed via Gaussian kernel over 0.5 s in each frequency, and then averaged over all animals for visualization and analysis.

### Phase analyses

The phase locking analyses methods have been described previously in detail (Park et al., 2010a; Ahn et al., 2014). Briefly, signals were Kaiser windowed and digitally filtered using a FIR filter in the θ frequency band (5–11 Hz) at the sampling rate of 1017 Hz. Zero phase filtering was used to avoid phase distortions. Phase was extracted via Hilbert transform resulting in two signals; $\varphi_{1}\left(t\right)$ and $\varphi_{2}\left(t\right)$ (Pikovsky et al., 2001; Hurtado et al., 2004). To clarify, let x(t) be the filtered signal at the given frequency band. Then the complex analytic extension of x(t) is given by Eqn. 1

(1) $$\zeta \left(t\right)=x\left(t\right)+i\;\overset{¯}{x}\;(t)$$

where $\overset{¯}{x}\left(t\right)$ is given by the Hilbert transform of the signal (Eqn. 2)

(2) $$\overset{¯}{x}\left(t\right)=H\left(x\right)=\frac{1}{\pi }p.v.\int_{-\infty }^{\infty }\frac{x(\tau )}{t-\tau }d\tau .$$

Then the analytic signal is projected on the unit circle (Eqn. 3)

(3) $$z\left(t\right)=\frac{\zeta \left(t\right)}{\left\|\zeta \left(t\right)\right\|}=e^{i\varphi (t)}$$

where $\left\|\zeta \left(t\right)\right\|$ is the modulus of $\zeta \left(t\right)$. The phase φ(t) was then extracted through the argument (angle) of $z\left(t\right).$

### Phase locking analyses

The following widely used measure of the strength of phase locking between these two signals was calculated: (Eqn. 4)

(4) $$\gamma =\left\|\frac{1}{N}\sum_{j=1}^{N}e^{i\theta \left(t_{j}\right)}\right\|$$

where $\varphi_{1}\left(t\right)$ and $\varphi_{2}\left(t\right)$ are two phases from the filtered signals, the phase difference $\theta \left(t_{j}\right)=\varphi_{1}\left(t_{j}\right)-\varphi_{2}\left(t_{j}\right)$, $t_{j}$ are the times of data points, and N is the number of all data points during the given time interval. The values of this phase locking index vary from 0 (no phase locking) to 1 (perfect phase locking). This kind of phase synchrony index has been shown to be appropriate to study neural oscillatory synchronization of widely varying strength (Lachaux et al., 1999; Pikovsky et al., 2001; Hurtado et al., 2004). To explore the changes of synchronized dynamics for each of the three events over the trials, we computed the phase synchrony index γ for three epochs (before CS, during US, after US) at each trial. A total of 33-s windows was used. CS on occurred at 10 s, US on started 3 s after CS on and lasted for 10 s, and US off started 10 s after the end of US on.

### Phase delay analyses

To investigate the impact of CS presentation on the phase of the signals, we analyzed the phase delay. Phase delay was calculated by first computing the averaged time difference between peaks of LFP phases for 1 s before CS on for each brain region (mPFC, NA). Then, the time difference between the first peak right after the CS and the last peak right before the CS was computed. Phase delay was derived by computing the difference between the first value and second value. Positive values indicate that the CS causes a delay of peak while negative values suggest that the CS causes the advance of the peak.

### Intertrial phase coherence (ITPC)

To measure the CS-evoked precision of phases across trials in the θ band, ITPC was calculated. Drinking trials were analyzed for 200 ms before, and 1 s after the CS. ITPC was calculated per Delorme and Makeig, 2004: (Eqn. 5)

(5) $$ITPC=\frac{1}{n}\sum_{k=1}^{n}\frac{F_{k}\left(f,t\right)}{\left|F_{k}\left(f,t\right)\right|}$$

After calculating ITPC for each trial, averages across trials were taken in the θ band (5–11 Hz) for each dataset. Statistics were performed on the dataset-averaged time series.

### Statistical analyses

All analyses were performed in MATLAB (MathWorks) and R (https://www.r-project.org/). Unless specified otherwise, all comparisons were first subjected to ANOVA testing or mixed-design ANOVA (a mixture of between subject factor and within subject factors) followed by *post hoc* tests for all multiple comparison procedures.

## Results

### Strain differences are observed in drinking and locomotor behavior

Consistent with our previous findings (McCane et al., 2014), behavioral differences were observed between strains during 2CAP recording sessions. P rats consumed more EtOH than Wistars (independent samples *t* test, *t*_(36)_ = 4.56, *p* = 5.70 × 10^−5^; Fig. 1*A*). However, P rats and Wistars did not differ in the percentage of drinking trials relative to non-drinking trials (independent samples *t* test, *t*_(36)_ = 9.71 × 10^−1^, *p* > 0.05; Fig. 1*B*). Additionally, strain differences in locomotor activity were observed. Movement velocity differed between P rats and Wistars, on both drinking (main effect of strain: *F*_(1,12073)_ = 15.5, *p* = 8.21 × 10^−5^) and non-drinking trials (main effect of strain: *F*_(1,13748)_ = 1972, *p* = 9.21 × 10^−6^), trial × strain interaction (*F*_(1,15792)_ = 2.90 × 10^1^, *p* = 7.46 × 10^−8^; Fig. 1*C*).

### Theta synchrony between mPFC-NA increases when P rats drink

On trials that animals drank, a robust change in voltage of the mPFC and NA was observed following the presentation of the CS in both strains of animals (Fig. 2). The change in voltage response was characterized most prominently by a fast negative going peak that occurred ∼150 ms after the presentation of the CS (Fig. 2*A*). Additionally, a slower positive going peak was observed between 4 and 5 s following the CS that roughly corresponded to the EtOH becoming available. These peaks were observed in both strains of animals and in both the mPFC and NA recordings (Fig. 2). However, these peaks were not as prominent during non-drinking trials (data not shown). P rats exhibited an enhanced response in the initial, negative going peak relative to Wistars in both the mPFC (strain × time interaction *F*_(300,162239)_ = 1.39, *p* = 1.05 × 10^−5^; Fig. 2*A*) and the NA (main effect of strain *F*_(1,162239)_ = 2.78 × 10^2^, *p* < 1.00 × 10^−16^; main effect of time *F*_(300,162239)_ = 3.15, *p* < 1.00 × 10^−16^; Fig. 2*B*).

Time-frequency and synchrony analyses were also performed on the voltage responses. Peaks in θ band were observed in the PFC and NA power spectra (Fig. 3*A*). Wavelet coherence was used to initially assess synchrony between the mPFC and NA (Fig. 3*B*,*C*), as it is sensitive to both phase and amplitude fluctuations and optimal for nonstationary signals (Lachaux et al., 2002). Increases in θ synchrony were observed between the mPFC and NA on drinking trials in both strains (Wistar, trial × time interaction, *F*_(4,170)_ = 6.75, *p* = 4.6 × 10^−6^; P rat, trial × time interaction, *F*_(4,190)_ = 6.82, *p* = 4.1 × 10^−6^). The increase in mPFC-NA synchrony was more robust in P rats (Fig. 3*D*).

**Figure 4.**
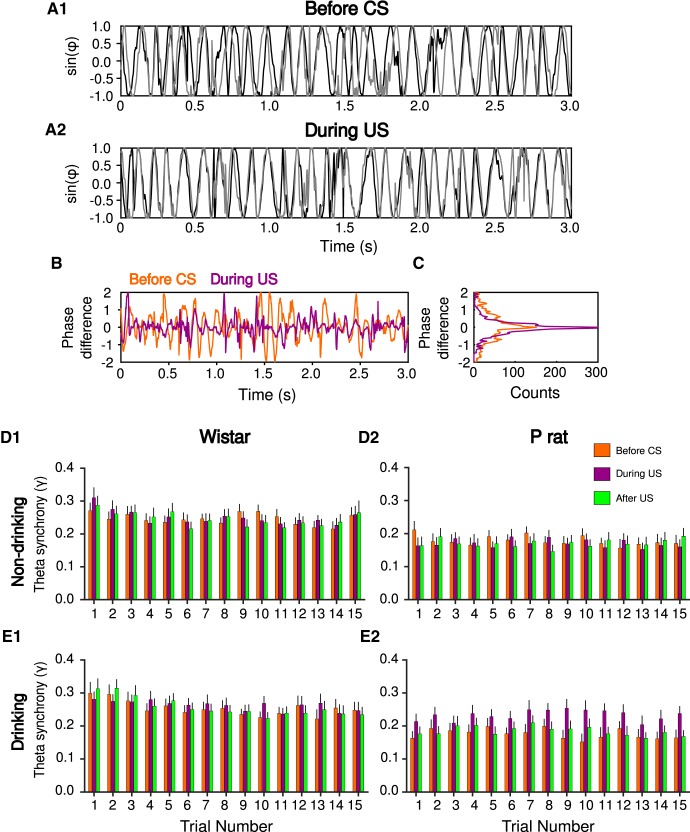
Physiologic differences are observed in the phase domain. Phase synchrony between the mPFC (black) and NA (gray) before drinking (***A1***) and during drinking (***A2***) indicate oscillations are more in phase during drinking, relative to before drinking. The difference in phase during (purple) and before (orange) drinking (***B***). Histogram of phase difference during drinking trials, notice more pronounced peak at zero indicating more synchronized phases (***C***). Theta band synchrony index (γ) for Wistar (***D1***, ***E1***) and P rats (***D2***, ***E2***) before the CS (orange), during the US (purple), and after the US (green) over 15 non-drinking (***D***) and drinking trials (***E***). Values are mean ± SEM.

Phase synchrony during the US but not before the CS was observed (Fig. 4*A–C*). To directly assess phase synchrony between the mPFC and the NA, the synchrony index (γ) was computed (Fig. 4*D*,*E*) for three behavioral epochs of the 2CAP (before the CS, during the US, and after the US) across the first 15 drinking and non-drinking trials. To investigate the trial by trial changes in γ, a mixed-design ANOVA [between subject factors strain (Wistar, P) and within subject factors epoch (before CS, during US, or after US), drinking status (drinking, non-drinking trials), and trial number (1–15)]. There was a significant main effect of drinking status (*F*_(1,21)_ = 1.39 × 10^1^, *p* = 1.26 × 10^−3^), indicating that, overall, synchrony differed for trials in which animals chose to drink versus trials where animals abstained.

Synchrony was next analyzed for drinking and non-drinking trials separately. A mixed-design ANOVA on non-drinking trials revealed a significant main effect of trial number (*F*_(14,378)_ = 2.42, *p* = 2.97 × 10^−3^; Fig. 4*D*). However, no effects of strain (*F*_(1,27)_ = 4.00, *p* > 0.05) or epoch (*F*_(2,54)_ = 2.63 × 10^−1^, *p* > 0.05) were observed. Additionally, no two-way or three-way interactions were detected (*p*s > 0.05). Collectively, these data indicate that, on non-drinking trials, the observed synchrony is influenced by trial number but not strain or epoch.

On drinking trials, significant main effects of strain (*F*_(1,29)_ = 4.64, *p* = 3.97 × 10^−2^), epoch (*F*_(2,58)_ = 1.50 × 10^2^, *p* = 5.45 × 10^−6^), and trial number (*F*_(14,406)_ = 4.61, *p* = 8.72 × 10^−8^) were observed. *Post hoc* tests indicated that phase synchrony for Wistars was significantly higher overall compared to P rats (Tukey’s HSD, *p* = 4.40 × 10^−2^; Fig. 4*E*). Also, synchrony during the US was significantly higher compared to a baseline period that occurred immediately before the CS (Tukey’s HSD, *p* = 6.16 × 10^−4^) and immediately after the sipper was removed from the chamber (Tukey’s HSD, *p* = 4.01 × 10^−3^). Additionally there were significant strain × epoch (*F*_(2,58)_ = 1.10 × 10^1^, *p* = 8.69 × 10^−5^), and strain × trial (*F*_(14,406)_ = 2.89, *p* = 3.52 × 10^−4^) interactions, indicating the pattern of phase synchrony across trials and epochs was different across rat strain. Therefore, drinking trials were analyzed separately for Wistar and P rats to determine differences in synchrony across each epoch and trial.

In Wistars, synchrony decreased across drinking trials (main effect of trial number, *F*_(14,182)_ = 5.18, *p* = 3.48 × 10^−8^), but neither a main effect of epoch (*F*_(2,26)_ = 1.76 × 10^−1^, *p* > 0.05) nor an epoch × trial interaction (*F*_(28,364)_ = 1.33, *p* > 0.05) were observed (Fig. 4*E1*). These data indicate that, in Wistars, while synchrony changes across drinking trials, it is not influenced by epoch.

In P rats, synchrony did not change across drinking trials (main effect of trial number, *F*_(14,224)_ = 1.40, *p* > 0.05). However, synchrony differed by epoch (main effect of epoch, *F*_(2,32)_ = 2.62 × 10^1^, *p* = 1.82 × 10^−7^; Fig. 4*E2*). *Post hoc* tests indicated that synchrony during drinking (US) was significantly higher than synchrony before CS presentation (Tukey’s HSD, *p* = 1.43 × 10^−4^) and after drinking (Tukey’s HSD, *p* = 3.27 × 10^−4^). There was no difference in synchrony before CS on and after drinking (Tukey’s HSD, *p* > 0.05). In summary, consistent with wavelet coherence analyses, increases in θ band synchrony were observed when alcohol was available during drinking trials (Fig. 4). The time scale of increases in drinking suggest that increases in the θ synchrony correspond to when P rats are consuming alcohol since the increase was consistently observed over trials during the drinking epoch.

### Presentation of the CS affects oscillatory dynamics

**Figure 5.**
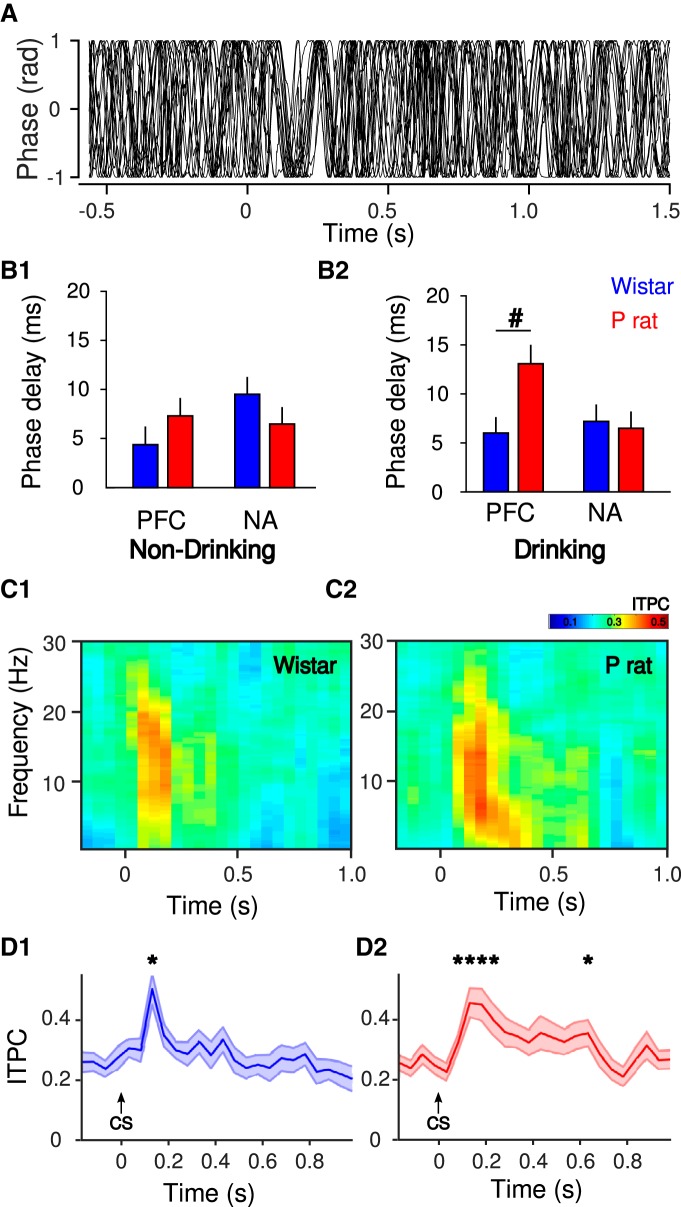
A pronounced phase reset is observed in the mPFC following presentation of the CS (***A***). MPFC and NA phase delay duration during non-drinking (***B1***) and drinking (***B2***) trials in Wistar (blue) and P rats (red). During non-drinking trials, there was no effect of the CS on the phase of the oscillation (***B1***). During drinking trials, P rats exhibit a greater CS-evoked phase delay in the mPFC, relative to Wistars (***B2***). There was no effect of the CS on phase delay in the NA. ITPC spectrograms (***C***) and phase coherence (***D***) for Wistar (1) and P rats (2). Both strains exhibit increases in ITPC following the CS; #*p* < 0.05, main effect of strain; *p < 0.05, Bonferroni *post hoc*, time different from baseline (200 ms before CS).

To determine how the presentation of the CS influenced oscillatory dynamics, changes in θ phase over time in the mPFC and NA were assessed (Fig. 5). To determine how the CS affects the phase of the signals, we assessed the phase-response curve. This measures the time difference between peaks of an oscillation, thus providing an index of phase precision within a trial and a way to detect phase resets. Phase-response curves were computed for the θ filtered LFP signal around the CS for each brain region (mPFC and NA separately). In both Wistars and P rats, the mean phase-response curve values for both brain regions were positive, indicating that presentation of the CS is associated with increases in the latency to the next peak of the θ phase following the CS thus indicating a phase reset (Fig. 5*A*,*B*). To further investigate the CS-evoked phase reset, a mixed-design ANOVA (between subject factor strain and within subject factors brain region and trial numbers) was performed. During drinking trials, there was a significant main effect of strain (*F*_(1,29)_ = 4.37, *p* = 4.54 × 10^−2^) but not brain region (*F*_(1,29)_ = 3.48, *p* > 0.05) or trial number (*F*_(14,406)_ = 5.97 × 10^−1^, *p* > 0.05). Both two-way and three-way interactions were not significant (*p*s > 0.05). Next, the difference of the phase delays was assessed for both strains in each brain region separately. In the mPFC, the phase delay of P rats was significantly longer compared to Wistars (Tukey’s HSD, *p* = 3.52 × 10^−2^; Fig. 5*B*). However, there was no difference between Wistar and P rats in the NA (Tukey’s HSD, *p* > 0.05). During non-drinking trials, a mixed-design ANOVA was employed to investigate the change in the phase of the signals due to CS presentation. There was no main effect of strain (*F*_(1,28)_ = 1.36 × 10^−1^, *p* > 0.05), brain region (*F*_(1,28)_ = 1.51 × 10^−1^, *p* > 0.05) or trial number (*F*_(14,392)_ = 1.39, *p* > 0.05; Fig. 5*B1*). Both two-way and three-way interactions were not significant (*p*s > 0.05). Collectively these analyses indicate that the phase resets are most robust in P rats in the mPFC on drinking trials.

Stimulus evoked correlations in neural activity have been suggested to play a role in encoding of environmental stimuli and neural plasticity (Mazzoni et al., 2008). To determine if the phase resets detected in the previous analyses lead to stimulus-evoked synchrony in the phases of mPFC LFP’s, ITPC was assessed across drinking and non-drinking trials. Transient increases in ITPC were observed in both Wistars (repeated-measures ANOVA, main effect of time, *F*_(23,391)_ = 3.02, *p* = 5.50 × 10^−6^) and P rats (repeated-measures ANOVA, main effect of time, *F*_(23,437)_ = 3.73, *p* = 0.1 × 10^−6^). CS-evoked changes in ITPC were assessed by comparing ITPC values 200 ms before stimulus onset to values 1 s after CS presentation (Fig. 5*D*). Wistars exhibited increases in ITPC 50 ms following the CS, whereas P rats exhibited increases for 200 ms following ITPC (Fig. 5*D*). Collectively these data indicate that CS-evoked increases in ITPC are observed in both strains of animals.

### Tolcapone reduces EtOH intake and number of drinking trials in P rats

**Figure 6.**
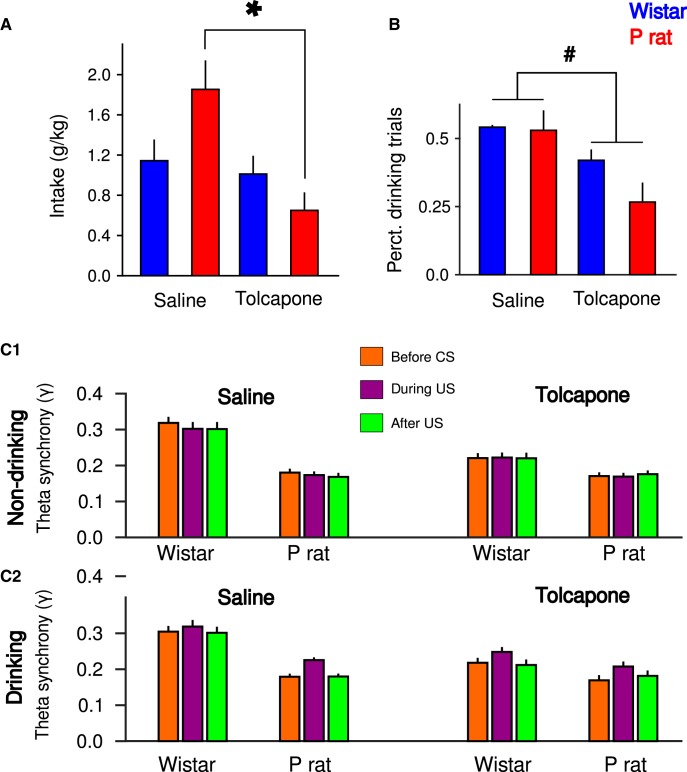
The behavioral and physiologic effects of tolcapone. Tolcapone decreases EtOH intake (***A***) in P rats but not Wistars and number of drinking trials in both strains (***B***). Tolcapone reduces mPFC-NA synchrony in Wistars (***C***); *p < 0.05, Bonferroni *post hoc*, main effect of treatment in P rats; #*p* < 0.05 main effect of treatment.

Following treatment with either saline or tolcapone, the amount of EtOH consumed and the number of drinking trials were assessed. For intake, there was a significant main effect of treatment (*F*_(1,22)_ = 1.47 × 10^1^, *p* = 9.01 × 10^−4^) but no effect of strain (*F*_(1,22)_ = 7.23 × 10^−1^, *p* > 0.05). There was also a significant strain × treatment interaction (*F*_(1,22)_ = 4.39, *p* = 4.79 × 10^−2^). *Post hoc* test indicated that tolcapone decreased intake of EtOH (Tukey’s HSD, *p* = 9.01 × 10^−4^). In particular, tolcapone decreased intake in P rats (Tukey’s HSD, *p* = 1.35 × 10^−3^) but not Wistars (Tukey’s HSD, *p* > 0.05; Fig. 6*A*). For the percentage of drinking trials, a significant main effect of treatment (*F*_(1,18)_ = 6.19, *p* = 2.28 × 10^−2^) was observed, but no main effect of strain (*F*_(1,18)_ = 1.14, *p* > 0.05) or strain × treatment interaction (*F*_(1,18)_ = 8.39 × 10^−1^, *p* > 0.05) were observed. *Post hoc* testing indicated that treatment with tolcapone resulted in fewer drinking trials compared to treatment with saline (Tukey’s HSD, *p* = 2.28 × 10^−2^; Fig. 6*B*).

### Tolcapone attenuates mPFC-NA synchrony

In the following analyses, data are collapsed across trial number due to the few drinking trials observed following tolcapone treatment in P rats. There was no effect of tolcapone on CS-evoked changes in voltage (Fig. 2) in either strain or brain region (*p*s > 0.05; data not shown). To investigate the effects of tolcapone on mPFC-NA synchrony (γ), we performed a four-way ANOVA with treatment (saline or tolcapone), strain, epoch, and drinking status (drinking or non-drinking trials) as factors. There were significant main effects of treatment (*F*_(1,1635)_ = 3.16 × 10^1^, *p* = 2.27 × 10^−8^), strain (*F*_(1,1635)_ = 1.72 × 10^2^, *p* < 1.0 × 10^−16^), and drinking status (*F*_(1,1635)_ = 5.24, *p* = 2.21 × 10^−2^), but not epoch (*F*_(2,1635)_ = 1.47, *p* > 0.05). There was also a significant treatment × strain interaction (*F*_(1,1635)_ = 2.83 × 10^1^, *p* = 1.17 × 10^−7^), while all other two-way, three-way, or four-way interactions were not significant (*p*s > 0.05). *Post hoc* tests indicated that administration of tolcapone resulted in reduced mPFC-NA synchrony (Tukey’s HSD, *p* = 1.95 × 10^−8^; Fig. 6*C*), an effect driven by a tolcapone mediated reduction in Wistars (Tukey’s HSD, *p* = 1.06 × 10^−10^) but not P rats (Tukey’s HSD, *p* > 0.05). Consistent with non-treatment sessions, overall mPFC-NA synchrony in Wistars was higher compared to P rats (Tukey’s HSD, *p* = 1.67 × 10^−10^). Moreover, mPFC-NA synchrony during drinking trials was significantly higher than during non-drinking trials (Tukey’s HSD, *p* = 2.20 × 10^−2^).

### Tolcapone reduces CS-evoked phase delays in P rats

**Figure 7.**
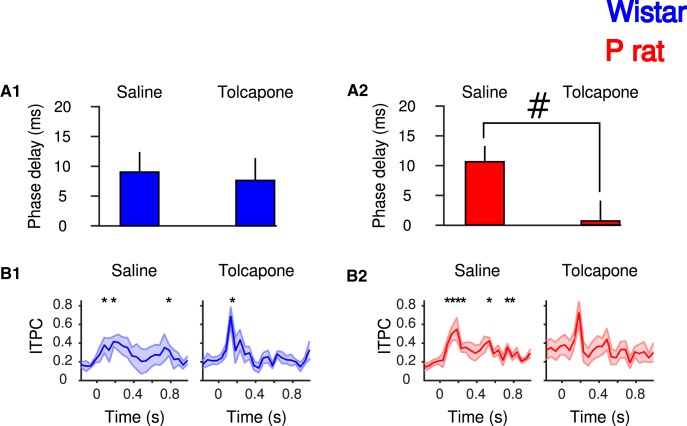
The effects of tolcapone on the phase delay in Wistar (***A1***; blue) and P rats (***A2***; red) in the mPFC. Mean phase coherence (***B***) in Wistar (left) and P rats (right) under saline or tolcapone treatment. Tolcapone reduces the phase delay in P rats but not Wistars (***A2***). Tolcapone results in increased phase coherence following the CS in Wistars (***B1***) but not P rats (***B2***); #*p* < 0.05 Tukey’s HSD *post hoc*; *p < 0.05, time different from baseline.

Phase-response curves were computed to assess CS-induced phase resets following treatment and were collapsed across trials. A four-way ANOVA (factors: treatment, strain, drinking status, brain region) was performed. There was a significant main effect of strain (*F*_(1,1168)_ = 7.31, *p* = 6.96 × 10^−3^). *Post hoc* testing indicated that the phase delays of P rats were significantly shorter compared to Wistars (Tukey’s HSD, *p* = 6.86 × 10^−3^; Fig. 7*A*). There were no other main effects of treatment (*F*_(1,1168)_ = 3.07 × 10^−1^, *p* > 0.05), drinking (*F*_(1,1168)_ = 1.37, *p* > 0.05) or brain region (*F*_(1,1168)_ = 2.30 × 10^−1^, *p* > 0.05). However, there was a significant treatment × strain interaction (*F*_(1,1168)_ = 4.24, *p* = 3.97 × 10^−2^). *Post hoc* testing indicated that tolcapone reduced the phase delay in the mPFC of Prats during drinking trials (Tukey’s HSD, *p* = 7.48 × 10^−3^; Fig. 7*A2*); an effect not observed during non-drinking trials, in Wistars or in the NA (*p*s > 0.05). Similar to non-treatment sessions, a main effect of strain was observed (*F*_(1,464)_ = 1.08 × 10^1^, *p* = 1.08 × 10^−3^) with *post hoc* test indicating a shorter phase delay in P rats, relative to Wistars (Tukey’s HSD, *p* = 1.00 × 10^−3^).

To further determine how changes CS-evoked synchrony were influenced by treatment, ITPC was assessed following saline and tolcapone injections on drinking trials. In Wistars, no main effect of time was observed following saline administration (repeated-measures ANOVA, *F*_(23,69)_ = 1.09, *p* = 0.37), whereas a main effect of time was observed following tolcapone administration (repeated-measures ANOVA, *F*_(23,92)_ = 3.00, *p* = 1.04 × 10^−4^; Fig. 7*B*). In P rats a main effect of time was observed following saline (repeated-measures ANOVA, *F*_(23,115)_ = 2.95, *p* = 7.41 × 10^−5^), but not tolcapone (repeated-measures ANOVA, *F*_(23,161)_ = 1.30, *p* = 0.178).

## Discussion

These experiments investigated the neural mechanisms by which drug paired stimuli elicit reward seeking in a rodent model of addiction vulnerability. Relative to Wistars, P rats exhibited an increase in mPFC-NA synchrony when drinking and a greater CS-induced phase delay in mPFC. Following tolcapone administration, a reduction in EtOH intake in P but not Wistar rats was observed. While no effect of tolcapone on synchrony in P rats was observed, a tolcapone-mediated suppression of the mPFC phase delay was observed in P rats but not Wistars during drinking trials only. These data suggest a novel neural mechanism whereby tolcapone suppresses drinking in genetically susceptible populations. These data also further elucidate the modulatory role of catecholamines in the corticostriatal system on alcohol seeking behaviors.

### Corticostriatal synchrony is associated with drug-seeking phenotypes

Theta oscillations in the mPFC are hypothesized to play a critical role in encoding reward value. MPFC θ phase locking was shown to be present during both reinforced and non-reinforced licks in a high-value context while phase locking was notably weaker during both reinforced and non-reinforced licks in a low-value context, suggesting that the strength of mPFC θ activity corresponds to reward value (Amarante et al., 2017). Stronger frontal cortex θ phase locking was observed in anticipation of sucrose compared to quinine (van Wingerden et al., 2010b), further suggesting θ oscillations encode subjective value of a reward.

In the present experiment, P rats exhibited lower mPFC-NA θ synchrony than Wistars overall (Fig. 4*D*,*E*). Low synchrony may reflect impairments in corticostriatal connectivity and contribute to the excessive drinking phenotype of the P rat. Reduced corticostriatal synchrony is associated with impaired executive function in AUD (Courtney et al., 2013). Moreover, deficits in corticostriatal connectivity are hypothesized to result in a reduction in inhibitory control of the mPFC over the striatum, resulting in poor control over behavioral responding and, possibly, a precursor to compulsive behaviors (Forbes et al., 2014). Poor frontal-striatal synchrony is also associated with alcohol craving (Park et al., 2010b) and dependence severity (Courtney et al., 2013). While the current study is not able to differentiate whether strain differences in corticostriatal synchrony are a consequence of alcohol exposure, genetic background, or an interaction of the two, human imaging studies suggest that impaired connectivity may be associated with genetic vulnerability to addiction disorders. Alcohol naïve youth with a family history of alcohol showed reduced corticostriatal synchrony relative to youths with no family history (Cservenka et al., 2014). FH+ individuals also exhibit reduced cortical activation during behavioral inhibition (Schweinsburg et al., 2004) and greater frontal response to alcohol paired stimuli (Kareken et al., 2010), which further suggests that altered activity in the PFC and striatum may be associated with genetic vulnerability for addiction. Importantly, these data support the hypothesis that impaired frontal-striatal connectivity may be an endophenotype for alcohol addiction and lend further credibility to the use of the P rat as a translational model of AUD symptoms.

Alcohol is hypothesized to disrupt the structure of oscillations by making them asynchronous (Ehlers et al., 1998; Rangaswamy and Porjesz, 2014). Alcohol exposure decreases the prevalence of θ oscillations (Givens, 1995) and θ power (Ehlers et al., 1992). Because P rats typically consume more EtOH than Wistars, reduced overall mPFC-NA synchrony may be linked to differences in EtOH exposure. In line with this view, EtOH administration reduced phase locking to an auditory stimulus in both rats and humans and these EtOH-induced changes were correlated with blood EtOH concentrations and subjective measures of EtOH intoxication (Ehlers et al., 2012). However, while no differences in mPFC-NA synchrony were observed over trials in P rats, a decrease was observed in Wistars. Similarly, our group has shown that the effects of alcohol on neural firing in the mPFC are influenced by rat strain (Linsenbardt and Lapish, 2015). These findings add complexity to the perhaps too simplified view that the pharmacological effects of EtOH are disruptive to synchrony. Rather, these data indicate that effects of EtOH on mPFC-NA synchrony can be influenced by genetic background.

A wealth of literature supports the hypothesis that PFC-NA connections are important for reward seeking, especially in the presence of CSs (Stefanik et al., 2013; McGlinchey et al., 2016). In the current study, tolcapone administration led to modest reductions in mPFC-NA synchrony in Wistars but not P rats. In contrast, tolcapone reduced EtOH intake in P rats but not Wistars. These data suggest a dissociation between mPFC-NA synchrony and EtOH consumption and that increases in catecholamine efflux evoked by tolcapone do not prevent synchrony between the mPFC and NA. Moreover, these data further suggest that the critical site of action of tolcapone is in mPFC.

### Cue-evoked alterations in θ phase is associated with alcohol seeking in P rats

A robust θ phase reset was observed in mPFC following cue presentation (Fig. 5*B2*). Phase resets have been shown to provide a timing mechanism that ensures optimal processing of inputs coming into a brain region by temporally aligning an oscillation to enhance information processing (Lakatos et al., 2007; Rajkai et al., 2008; Melloni et al., 2009). They are often observed in response to exposure to a salient environmental stimulus. Furthermore, phase resets are associated with expression of conditioned responses (Courtin et al., 2014) and emerge as the relationship between a CS and response is learned (Taub et al., 2018). Presentation of a CS also results in an increase in amplitude and phase reset of θ oscillations (Courtin et al., 2014). Therefore, it is not surprising that a θ phase reset was observed following cue presentation in the current study and the phase reset is not likely specific to processing an alcohol-associated cue but, more generally, reflects an environmentally salient stimulus. However, the fact that the phase reset was more pronounced in P rats on drinking trials (Fig. 5*B2*) indicates that it contributes in some way to the motivating effects of the cue to elicit drinking and is possibly associated with an enhanced risk for excessive drinking.

### Tolcapone-mediated reduction of CS-evoked responses is associated with reduced EtOH consumption

Alcohol associated cues elicit increases in the frontal BOLD response (Oberlin et al., 2016) and the extent of activation of these regions is correlated with alcohol craving (Myrick et al., 2004). Stimuli paired with reward come to possess motivational properties (Robinson and Berridge, 2001) and are themselves capable of inducing drug wanting or craving in drug-dependent individuals (Reid et al., 2006; Vollstädt-Klein et al., 2012). In the experiments presented here, tolcapone administration blunted the CS-evoked phase reset in P rats, which may have led to a reduction in the motivational salience of the CS. In human subjects, tolcapone reduced reaction time and the ability of subjects to make the correct saccade following a visual cue (Cameron et al., 2018), suggesting that COMT inhibition may dampen salience attribution and subsequent attention to stimuli. We have previously reported null effects of tolcapone on free-choice drinking but reduced cued EtOH consumption, supporting the hypothesis that tolcapone-mediated changes in EtOH intake observed here are cue dependent (McCane et al., 2014). Therefore, we hypothesize that tolcapone-mediated disruption of the phase reset may contribute to blunted incentive motivational properties of the cue.

Strain differences in tolcapone’s ability to reduce the phase reset may be associated with differences in basal DA tone and subsequent differences in cue-evoked DA transmission. Alcohol-paired cues elicit increases in DA concentrations in both clinical (Volkow et al., 2006; Oberlin et al., 2013) and preclinical subjects with a history of drinking (Melendez et al., 2002). Repeated presentation of reward-paired stimuli can strengthen both cortical stimulus representation and neural responses to these stimuli, an effect enhanced when paired with DA efflux (Bao et al., 2001; Frankó et al., 2010). Tolcapone has no effect on basal DA tone but instead enhances evoked DA release (Tunbridge et al., 2004; Lapish et al., 2009). In P rats, tolcapone may diminish the influence that stimuli have over behavior by preventing the metabolism of cortical DA and increasing tone on DA auto receptors. The effect of increased DA would be blunted in Wistars who already exhibit greater levels of extracellular DA coupled with increased expression of COMT in mPFC and therefore a greater capacity to buffer changes in DA efflux (Engleman et al., 2006; McCane et al., 2018).

The results obtained here highlight the heterogeneity of factors which may contribute to excessive drinking phenotypes. Whereas alterations in corticostriatal synchrony may be associated with excessive drinking, catecholamine activity in the PFC appears to more strongly influence attribution of motivational salience to drug paired stimuli in addiction vulnerable organisms. These results highlight the role that catecholamines play on shaping how motivating stimuli are processed by PFC. Furthermore, these results suggest that targeting the catecholamine system in PFC may provide an effective way to blunt the incentive motivational properties of drug-associated stimuli. In this way, novel treatments that target this system might be effective in preventing craving and relapse.

## Synthesis

Reviewing Editor: Karen Szumlinski, University of California at Santa Barbara Decisions are customarily a result of the Reviewing Editor and the peer reviewers coming together and discussing their recommendations until a consensus is reached. When revisions are invited, a fact-based synthesis statement explaining their decision and outlining what is needed to prepare a revision will be listed below. Note: If this manuscript was transferred from JNeurosci and a decision was made to accept the manuscript without peer review, a brief statement to this effect will instead be what is listed below.

I have read this revised report, which was transferred from JNS. While lacking in direct cause-effect analyses, this report contains new and interesting information pertaining to the relationship between neural activity within the corticostriatal pathway and the propensity to drink. In my opinion, the authors have satisfactorily addressed the concern regarding the source of the animals used in the study and I found no other issues with this report as written. Thus, I am happy to accept this report for publication in eNeuro.
